# A Head-to-Head Comparison of Computed Tomography Colonography, Optical Colonoscopy, and Colon Endoscopic Capsule for the Detection of Polyps After Partial Colectomy or Rectosigmoidectomy for Colorectal Cancer: A Pilot Study

**DOI:** 10.7759/cureus.38410

**Published:** 2023-05-01

**Authors:** Christiano Makoto Sakai, Serli Kiyomi Nakao Ueda, Angela Hissae Motoyama Caiado, Igor Braga Ribeiro, Fabio Ramalho Tavares Marinho, Daniel Tavares de Rezende, Leonardo A Bustamante-Lopez, Sergio C Nahas, Diogo Turiani Hourneaux de Moura, Eduardo Guimarães Hourneaux de Moura

**Affiliations:** 1 Department of Gastroenterology, Faculty of Medicine, Hospital das Clínicas of the University of São Paulo, São Paulo, BRA; 2 Department of Radiology, Hospital das Clínicas of the University of São Paulo, São Paulo, BRA; 3 Department of Radiology, Faculty of Medicine of the University of São Paulo, São Paulo, BRA; 4 Endoscopy Service, Santa Casa de Misericórdia de Maceió, Maceió, BRA; 5 Endoscopy Service, Clinical Hospital of the Federal University of Goiás, Goiania, BRA; 6 Colorectal Surgery, Hospital das Clínicas of the University of São Paulo, São Paulo, BRA

**Keywords:** virtual colonoscopy screening, endoscopic capsule, endoscopy, cancer, colorectal

## Abstract

Background and objective

Optical colonoscopy is the gold standard method for the diagnosis of colorectal cancer (CRC) and it allows for biopsy and resection, as well as documentation of synchronous lesions. CT colonography (CTC) and colon endoscopic capsule (CEC) are also recommended as alternative minimally invasive or non-invasive procedures. Prospective studies comparing these three approaches are scarce in the current literature. In light of this, the aim of this pilot study was to compare the efficacy of polyp detection between these three methods in patients with a history of curative surgical resection of CRC.

Methods

Patients were consecutively recruited and all procedures were sequentially conducted on the same day. The primary endpoint was the detection rate of polyps, whereas secondary endpoints were the detection of polyps according to size and location, and the adverse events caused by these procedures.

Results

A total of 21 patients were consecutively included and all of them underwent all three interventions. No adverse events, local recurrences, or metachronous lesions were detected. In two cases with elevated carcinoembryonic antigen (CEA), CTC unveiled distant metastasis. Optical colonoscopy registered a mean of 0.4 polyp >6 mm and 1.3 polyps <6 mm per patient. CTC unveiled only 0.5 polyp >6 mm/patient and no smaller lesions were documented, whereas findings for the colon capsule comprised an average of 0.4 polyps >6 mm and 0.7 polyps <6 mm per patient. Statistical difference was not demonstrated, except for virtual colonoscopy in terms of the total number of polyps detected in comparison to optical colonoscopy.

Conclusions

Optical colonoscopy showed superior results in comparison to virtual colonoscopy while there was no statistical difference in comparison to colon capsule. Notwithstanding occasional difficulties, all three techniques were well tolerated. Hence, decisions concerning the use of each diagnostic method should be based on their availability, professional expertise, contraindications, and patient preferences.

## Introduction

According to the Global Cancer Statistics (GLOBOCAN), 1.9 million cases of colorectal cancer (CRC) were diagnosed in 2020 worldwide. It is the third most frequent malignancy in men and the second in women [1]. About 50% of CRC cases are localized (stages I and II), 25% are locally invasive (stage III), and the rest are associated with distant metastasis (stage IV) [2]. Screening programs for asymptomatic cases (primary prevention) as well as for operated patients (secondary prevention or surveillance) have been implemented in many countries in order to enable early detection, thereby reducing morbidity and mortality.

Optical colonoscopy is the gold standard for the diagnosis and follow-up of CRC because it allows for identification, biopsy, and resection of polyps, apart from addressing synchronous or metachronous tumors and other lesions. However, this procedure is not free of risks and can be costly, demanding a high level of professional expertise for safe and effective execution. CT colonography (CTC) or virtual colonoscopy employing multislice machines and reconstruction techniques can provide bidimensional and tridimensional images of the colon and rectal lumen, enabling the diagnosis of polyps and other mucosal abnormalities. As the whole abdomen is analyzed, pericolonic and extracolonic findings are also possible. Given that cancer risk in small polyps is low, articles usually target structures >6 mm [3]. It is already recommended as a diagnostic method by the American Cancer Society, the European Society of Endoscopy (ESGE), and the European Society of Gastrointestinal Radiology (ESGAR) in cases where optical colonoscopy is not feasible or insufficient [4]. The swallowable endoscopic capsule was developed in the 1990s for the study of small bowel lesions and is the gold standard for the investigation of obscure gastrointestinal bleeding. For large bowel lesions, a more advanced model was designed in 2009 (Pillcam 2®; Medtronic), encompassing two cameras and a longer operation time, compatible with full colonic visualization [5]. The protocol does not involve any sedation, gas insufflation, or radiation exposure, and it is minimally invasive. Although the investigation involves an all-day procedure, and polyethylene glycol and Phospho-soda ingestion are required for optimal results, there is a low risk of capsule impaction or non-elimination in cases of strictures or intestinal subocclusion.

Evidence suggests that optical colonoscopy, CTC, and colon endoscopic capsule (CEC) could all be useful in the diagnosis of polyps in postoperative patients who have undergone colectomy or rectosigmoidectomy due to CRC. However, no trial has been conducted employing all three modalities in this patient population for the purpose of comparison. Hence, an experiment involving a head-to-head, cross-over investigation was designed, in which each patient represented his or her own control. It was hypothesized that within a similar, well-controlled context, all three methodologies would be demonstrated as efficient and the selection should be based on availability, professional experience, intolerances or contraindications, as well as preferences or previous experiences of the patient.

## Materials and methods

Ethical aspects 

This protocol was approved by the CapPesq Research Ethics Committee, Hospital das Clínicas, University of São Paulo, São Paulo, Brazil (number 505210) and all patients signed informed consent.

Experimental design

This was a prospective head-to-head cross-over trial that employed three interventions. All procedures were consecutively conducted on the same day, with blinded analysis of the results, in order to minimize any bias concerning demographic features, comorbidities, professional team, equipment, colonic preparation, or interpretation of the results.

Inclusion and exclusion criteria

The inclusion criteria were as follows: patients older than 18 years of age recruited from among those who underwent intent-to-cure operations of partial colectomy or rectosigmoidectomy for CRC one to five years prior to the study.

The exclusion criteria were as follows: patients who had undergone a colostomy or any other gastrointestinal tract ostomy; those with swallowing and gastrointestinal disturbances such as Zenker's diverticulum, gastroparesis, history of stricture, intestinal obstruction, severe colitis or diverticulitis; patients with an allergy or intolerance to Phospho-soda, simethicone, metoclopramide, bisacodyl, mannitol or propofol; those with bleeding diathesis or use of anticoagulants; those with inguinal hernia containing the sigmoid colon or other contraindications to colonic insufflation; women of reproductive age (with or without pregnancy or lactation); patients with a cardiac pacemaker; those who refused to participate in the study or to sign the informed consent.

Primary and secondary endpoints 

The primary endpoint was to compare the detection rate of polyps of each method. Secondary endpoints were identification rates for polyps according to size (>6 mm or <6 mm) and location (left or right colon) as well as the safety of the procedures.

Recruitment procedure 

A list of all cases undergoing the three selected operations in the last five years was requested from the Coloproctology Service of our institution and, after the preliminary screening of the charts for inclusion and exclusion criteria, telephone interviews were conducted with the remaining patients. Additional exclusion criteria were applied and consent to participate was addressed; the selected patients were included and they signed the informed consent.

Statistical analysis 

Statistical analysis was conducted with the IBM SPSS Statistics v 25 software (IBM Corp., Armonk, NY); statistical significance was defined as p<0.05, and calculation of confidence interval (95% CI) was conducted whenever appropriate. Bland-Altman tests [6] were employed to compare the results achieved with the three diagnostic modalities. 

Precise sample size calculation was not possible, due to the nonavailability of previous three-method trials, particularly using the head-to-head, cross-over model and the selected surgical modalities. Nevertheless, considering the primary endpoint, a sample of 15 was estimated to be endowed with a power of 80% for an alpha error of 0.05 [7]. It is worth mentioning that in light of the absolute homogeneity of participants and controls, cross-over protocols are considered substantially more reliable than multi-arm trials, even with the perfect selection, blinding, and randomization.

Randomization and blinding

There was no patient randomization in this protocol as all participants underwent all three interventions. The order of the interventions was not randomized either, for practical reasons (institutional routines). CTC was thus conducted first, followed by CEC, and finally colonoscopy. 

Patients could not be blinded because the three procedures were remarkably different from each other. The same was true for the different specialists performing them, alternatively belonging to the Endoscopy Unit (colonoscopy and colon capsule) or to the Radiology Unit (CTC). However, none of these professionals were aware of the results of the other two examinations, and the final interpretation of the results was conducted by an independent member of the team not involved with protocol participants or executing professionals.

Colon preparation 

Diet, bisacodyl laxative, and 10% mannitol were used during a two-day routine, as described before [8]. 

Technical aspects of the procedures

*CTC Examination* 

Colonic insufflation (CO_2_) was performed by an electronic apparatus, and after the digital radiography confirmed adequate bowel distension, the 64-detector CT scanner (Discovery CT 750HD, GE Healthcare, Chicago, IL) was programmed. Iodine contrast was not administered, and images were processed using a Brilliance Philips Medical System (Philips, Amsterdam, Netherlands) or GE Healthcare workstation.

*CEC Routine* 

The sensor belt was positioned on the abdomen, and the patient swallowed the Pillcam 2® capsule (Given Imaging) with water or a simethicone solution. Capsule location was periodically monitored and the data recorder alerted towards metoclopramide and/or Phospho-soda ingestion, according to manufacturer recommendations, in order to ensure speedy and efficient progression of the capsule through the gastrointestinal tract. The examination was deemed completed when the capsule was eliminated through rectal evacuation.

*Colonoscopy* 

Colonoscopy was performed under propofol sedation by an experienced endoscopist, using the FUJINON EC-590ZW/L (FujiFilm, Tokyo, Japan) colonoscope. After recovery, patients were discharged home.

Data storage, sharing, and processing 

Safety precautions were adopted regarding patients' personal data privacy at all times. Data collection and analysis were conducted in a fully blinded fashion, preventing patient identification, in accordance with domestic and international data protection legislation.

## Results

Study population

From the list of 132 patients provided by the Coloproctology Service, 89 were eliminated after the preliminary screening of the charts based on inclusion and exclusion criteria. After interviews, 43 patients were selected and additional exclusion criteria were applied and refusals were elicited; ultimately, 21 patients were included and signed informed consent.

The mean age of the participants was 59.5 ± 5.0 years, and the majority of the population was female (76.2%, 16/21). Right hemicolectomy was the most frequent operation (52.3%, 11/21), followed by rectosigmoidectomy (33.3%, 7/21) and left hemicolectomy (14.3%, 3/21). Of the 21 patients, 14 (66.6%) had already undergone post-surgery colonoscopy and seven (33.4%) were being examined for the first time. Carcinoembryonic antigen (CEA) was normal in the entire cohort, with the exception of two stage-IV patients, who were subsequently found to have distant metastases. Admission surgical staging unveiled few early cases (2/21 stage 0 and 2/21 stage I, each accounting for 9.5%); the rest had stage II (6/21, 28,5%), stage III (7/21, 33.3%), and stage IV (4/21, 19.0%) disease. Adjuvant chemotherapy was prescribed to 16/21 (76.1%) patients. However, no patient was treated with neoadjuvant drugs.

General findings

The preparation of the colon, as described above and adopted in our institution for many decades, with diet, laxatives, and 10% mannitol was satisfactory in all cases.

Based on optical colonoscopy, only one anastomosis presented abnormality, which was subsequently shown to be benign according to the histopathological study of the biopsies. This abnormality in the anastomosis was also detected in CTC and CEC. Minor differences concerned the anastomotic line, such as ileocolic and colocolic anastomosis, which could not be identified in 2/21 (9.5%) of CTC and 4/21 (19.0%) of CEC examinations. Statistically, these discrepancies were not confirmed as methodological differences. Intraluminal metachronous tumors were not detected.

Polyp yield 

**Figure 1:**
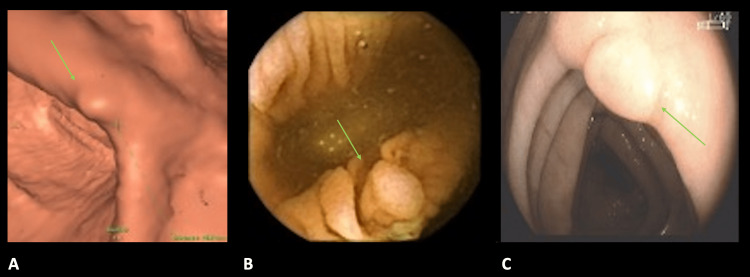
Polyps detected by different methods A. Computed tomography colonography (CTC). B. Colon endoscopic capsule (CEC). C. Optical colonoscopy

Optical colonoscopy identified a total of 36 polyps, comprising 27 <6 mm and nine >6 mm, besides the anastomotic abnormality already alluded to. All polyps were resected: by endoscopic mucosal resection (EMR) in three cases and by polypectomy snare or biopsy forceps in the rest. The biopsy of the irregular ileocolic anastomosis lesion displayed inflammatory tissue only. The majority of the polyps (21/36, 58.3%) were represented by tubular adenoma with low-grade dysplasia, and the morphological aspect of one of them was described as a laterally spreading tumor (LST). Another 12 polyps (33.3%) were hyperplastic, and the remaining three (8.3%) were inflammatory polyps. Local recurrences were not identified by any of the three techniques (Figure 1).

Differences between diagnostic methods were observed in terms of the number of identified polyps, which was statistically lower for CTC (11 polyps), but not for CEC (23 polyps). Optical colonoscopy identified an average of 0.4 polyps >6 mm and 1.3 <6 mm per patient. During CTC, 0.5 polyps >6 mm/patient were detected and no smaller lesions were considered, whereas findings for the capsule comprised an average of 0.4 polyps >6 mm and 0.7 polyps <6 mm per patient (Table 1).

**Table 1:** Differences in polyp yield in virtual colonoscopy (CTC) and colon endoscopic capsule (CEC), adopting optical colonoscopy as reference (100%) ^1^Evaluation of adenoma detection was not performed for CTC and CEC.^ 2^No <6 mm polyps were considered using CTC CTC: computed tomography colonography; CEC: colon endoscopic capsule

	Optical colonoscopy		CTC			CEC		
	N	%	N	%	P-value	N	%	P-value
Total polyps	36	100	11	30.6	0.037	23	63.9	0.207
Adenomas	21	100	-^1^	-	-	-^1^	-	-
<6 mm	27	100	-^2^	-	-	15	55.6	0.128
>6 mm	9	100	11	122.2	0.973	8	88.9	0.784
Right colon	16	100	3	18.7	0.004	2	12.5	0.002
Left colon	20	100	8	40.0	0.036	21	105	0.925

As expected, extracolonic lesions (liver and lung metastasis in two patients with elevated CEA) were only recognized in CTC.

Bland-Altman analysis showed no statistically significant difference between the three methods regarding the total number of polys and the size or location of the polyps. The only exception was the comparison between the total number of polyps detected in colonoscopy and CTC, which showed an average of 1.19 extra diagnoses of polyps in colonoscopy (p<0.01) (Table 2).

**Table 2:** Bland-Altman limits of agreement for the three methods *Student's t-test CTC: computed tomography colonography; CEC: colon endoscopic capsule

Variable	Techniques	Bias (95% CI)	Standard deviation	P-value*
Total polyps	Colonoscopy x CTC	1.19 (0.36–2.03)	1.83	<0.01
	Colonoscopy x CEC	0.57 (-0.55–1.69)	2.46	0.300
Polyps >6 mm	Colonoscopy x CTC	-0.05 (-0.47–0.37)	0,92	0.81
	Colonoscopy x CEC	0.10 (-0.19–0.38)	0.62	0.44
Polyps <6 mm	Colonoscopy x CEC	0.48 (-0.68–1.63)	2.54	0.55
Right-side polyps	Colonoscopy x CTC	0.67 (-0.04–1.38)	1.56	0.239
	Colonoscopy x CEC	0.76 (-0.14–1.66)	1.97	0.16
Left-side polyps	Colonoscopy x CTC	0.55 (0.06–1.04)	1.05	0.73
	Colonoscopy x CEC	-0.19 (-0.61–0.23)	0.93	0.223

Patient satisfaction survey

A questionnaire addressing patient experience with colonoscopy, CTC, and CEC was conducted. CEC is a minimally invasive method and was well accepted, but most patients preferred colonoscopy (15/21, 75.1%) performed under sedation, and six (25%) had no preference. One patient complained that staying awake during CTC was associated with some pain and discomfort during CO_2_ insufflation.

## Discussion

After intent-to-cure partial colon or colorectal resection, as in the present series, most centers enroll CRC patients in a five-year surveillance program. The fundamental aim is to manage metachronous lesions, as well as local and distant metastasis. The risk of recurrence reaches up to 30% for stages I-III [9,10] and up to 65% for those in stage IV [11,12]. In the current series, only 2/21 (9.5%) exhibited high concentrations of CEA, both of which were diagnosed with distant metastasis on CTC evaluation. Intraluminal cancer was not detected in any instances, not even in those two cases. In a long-term follow-up study of 10,283 patients with CRC, a comparatively low 0.3% annual incidence of metachronous cancer was unearthed [13]. Cumulative incidence after three years was 1.1%, 2.0% after six years, and 3.1% after 10 years. It is worth emphasizing that the majority of those patients had just one hemicolon, on account of previous surgical resection, suggesting that with an intact colon, more significant numbers would probably be encountered.

Conventional colonoscopy is the standard follow-up tool for this population, along with cancer antigen markers and general findings. In the current study, patients underwent colonoscopy one year postoperatively and then at intervals of three years. However, in cases displaying polyps, yearly examinations are scheduled at our institution. 

As alluded to in the Results section, 36 polyps were identified by conventional colonoscopy in the entire cohort, comprising nine polyps >6 mm and 27 polyps <6 mm. The primary endpoint was statistically higher for colonoscopy than the one observed with CTC, but not CEC. Other features such as the size or location of the polyps (right colon vs. left colon) were not statistically different. CTC seemed less useful than colonoscopy regarding the total of <6 mm polyps because according to consensus [3], these lesions were not considered. Interestingly, it identified larger polyps (>6 mm) at a higher rate, even though the advantage was not statistically confirmed. It was also able to document extracolonic metastasis.

In a meta-analysis by Porté et al. [14], CTC was 95% sensitive and 100% specific for anastomotic recurrence of CRC. It was also satisfactory in the detection of metachronous cancer. Information about polyps was not available, as the focus of the review was tumor recurrence only. In a comparative prospective trial by Weinberg et al. [15], 231 patients operated on for CRC underwent surveillance colonoscopy and CTC on the same day. The sensitivity of CTC was 44.0% for polyps ≥6 mm and 76.9% for those >10 mm, showing the advantage of colonoscopy.

The results of our study are different from those of the meta-analysis mentioned above [14]. One possible explanation is inadequate insufflation in patients lacking the ileocecal valve after right hemicolectomy, as gas would escape to the small bowel. In the same series, 42.6% of responding patients would prefer to undergo colonoscopy in the future, 35.4% had no preference, and only 22.0% preferred CTC the most, which is similar to our results. Two large European societies restrict CTC, with contrast injection, to those patients who prefer such an option or when colonoscopy is not feasible or contraindicated [16].

CEC has also been advocated as a legitimate tool for CRC surveillance and has been compared to colonoscopy and CTC [17-20]. This technique is minimally invasive and does not entail ionizing radiation, sedation, or CO_2_ insufflation, in contrast with the other two alternatives. Even though there are those mentioned major advantages, the CEC does involve a number of contraindications, particularly regarding swallowing derangements, intestinal stricture or subocclusion, and cardiac pacemakers. It also takes more time and involves a day-long program. The most feared complication is capsule retention, which could require endoscopy or even emergency surgery. However, this is an exceedingly rare event when patients are correctly screened. For doubtful cases, a sham absorbable capsule containing barium sulfate has been devised. The patient under suspicion swallows it, and gastrointestinal progress can be easily monitored as the device is radiopaque. In case of non-elimination after a reasonable period, the technique will be contraindicated and a plain abdominal X-ray will pinpoint the site of stricture or obstruction. Retrieval is not necessary as the sham capsule will be degraded by digestive secretions [21].

Direct comparisons with colonoscopy regarding polyp identification are scarce. The Deding study [17] provided quite substantial evidence in a comparison with CTC. CEC and CTC were consecutively conducted with the same method of colon preparation, as in our study. However, this was done one day after the other, not on the same day, and the aim was not a triple comparison, just CEC vs. CTC analysis. Among those patients who completed both procedures (there were incomplete CEC cases), the sensitivity of CEC was remarkably higher, notably for smaller polyps (6-9 mm).

On the basis of this and other studies, a huge population-based screening protocol has been announced in Denmark. Over 120,000 individuals with CRC risk demonstrated by the positive immunochemical fecal test will be submitted to CEC and subsequently confirmed by colonoscopy [21]. Despite such encouraging results, CEC is still not strongly recommended by the ESGE/ESGAR directive, with the evidence being qualified as moderate only [16].

This study has some limitations, and it primarily entails its design as a pilot study, notably the fact that a relatively small number of patients were involved, all in the postoperative period of CRC surgery. On the other hand, 66.6% of the patients had already undergone post-surgery colonoscopy at least three years before. This renders the results preliminary, requiring confirmation in larger studies. Also, some methodological details, such as the non-use of intravenous contrast medium during CTC, are not in line with other studies. The main strength of the protocol is the robust experimental design, with blinding of staff and investigators as much as feasible, and rigorous head-to-head, cross-over single-day comparison of the techniques. Thus, all candidates underwent all three procedures, each patient acting as his own control, with the same method of colon preparation and identical medical care.

All things considered, and given the lack of statistical significance for most of the findings in the Bland-Altman assessment, it can be affirmed that technological evolution is converging all three methods towards similarly high levels of performance, safety, and convenience. All are imaging procedures, even though each one is based on different physical and physiological premises and specific image-capturing devices. In this sense, it stands to reason that they will not be strictly regarded as competitors, but as complementary techniques to be made available at all large-volume services, indicated in accordance with the characteristics and clinical findings of each patient.

## Conclusions

Colonoscopy was demonstrated to be statistically superior to CTC, even though no significant advantage was found in comparison to CEC, and no statistical differences concerning identified polyp size or location were noticed. The biggest advantage of conventional colonoscopy is that it is a diagnostic procedure that offers therapeutic benefit in a single procedure, which is quite an advantage to the patient and hence is believed to be the preferred method of choice. All interventions were safe and well tolerated. Selection of a particular method should be based on availability, professional expertise, clinical features, and contraindications, as well as patient preference.
